# P05.10. Comparison of paper surveys and computer-assisted telephone interviews in a randomized controlled trial of yoga for low back pain

**DOI:** 10.1186/1472-6882-12-S1-P370

**Published:** 2012-06-12

**Authors:** C Cerrada, J Weinberg, D Dresner, A Boah, K Sherman, R Saper

**Affiliations:** 1 Boston Medical Center, Boston, USA; 2 Boston University School of Public Health, Boston, USA; 3 Group Health Research Institute, Seattle, USA

## Purpose

Little is known about the reliability of different forms of survey administration in CAM trials of low back pain. This analysis was designed to determine the reliability of responses to self-administered paper surveys and computer-assisted telephone administered interviews for pain intensity and the Roland Morris Disability Questionnaire among participants enrolled in a study of yoga for chronic low back pain.

## Methods

Forty-five of 95 participants enrolled in a 12 week dosing trial of yoga for chronic low back pain were randomly chosen to complete a telephone survey within 48 hours of completing a paper questionnaire. Both formats contained identical questions and data were collected at baseline, 6, and 12 weeks. Responses to an 11-point numerical rating scale for average low back pain intensity in the previous week and the modified Roland Morris Disability Questionnaire from both paper and telephone surveys were compared at baseline using the intra-class correlation coefficient (ICC). In addition, means and standard deviations were computed.

## Results

Preliminary analyses of paper survey and telephone interview responses for pain intensity and Roland Morris were compared for 44 participants who completed both baseline survey formats; one participant completed the paper survey but not the telephone interview. The means for pain intensity were 6.8 (SD=1.9) for the paper survey and 6.7 (SD=1.8) for the telephone interview. The means for the Roland Morris score were 12.7 (SD=5.3) for the paper survey and 13.2 (SD=5.9) for the telephone interview. The ICC for pain intensity and the Roland Morris were 0.86 and 0.89, respectively.

## Conclusion

Computer-assisted telephone interviews show excellent reliability as compared to traditional self-administered paper surveys in a low back pain yoga trial. Having two reliable options for data collection available may be helpful to increase response rates for principal outcomes in back pain trials.

